# Supplementary material for: Rigorous Validation of Paw Preference Using Three Complementary Behavioral Assays in Sprague Dawley Rats
Source: bioRxiv. 2026 Feb 10:2026.02.08.704691. Preprint. [Version 1] doi: 10.64898/2026.02.08.704691 (PMC12918835; doi:10.64898/2026.02.08.704691)
Supplement: 4 [file NIHPP2026.02.08.704691v1-supplement-4.pdf]

bioRxiv preprint doi: https://doi.org/10.64898/2026.02.08.704691; this version posted February 10, 2026. The copyright holder for this preprint (which was not certified by peer review) is the author/funder, who has granted bioRxiv a license to display the preprint in perpetuity. It is made available under aCC-BY 4.0 International license.

Appendix:

Supplemental Table S1. Summary of Rat Cohorts and Tests Administered:

Cohort	n	Age Range	Sex Distribution	Tests Administered
Core Inter-Test Group	30	12–48 wks	20 females, 10 males	Collins, Staircase, PaTRaT
Young Age Comparison	90	6–9 wks	Mixed	Collins Test only
Older Age Comparison	83	12–36 wks	Mixed	Collins Test only
Male Sex Comparison	93	6–48 wks	All male	Collins Test only
Female Sex Comparison	80	6-48 wks	All female	Collins Test only

This manuscript has been submitted for publication in Behavioral Brain Research. It has not yet undergone peer review.
